# Genetic Diversity and Natural Selection of *Plasmodium vivax* Duffy Binding Protein-II From China-Myanmar Border of Yunnan Province, China

**DOI:** 10.3389/fmicb.2021.758061

**Published:** 2021-11-29

**Authors:** Tian-Qi Shi, Hai-Mo Shen, Shen-Bo Chen, Kokouvi Kassegne, Yan-Bing Cui, Bin Xu, Jun-Hu Chen, Bin Zheng, Yue Wang

**Affiliations:** ^1^National Institute of Parasitic Diseases, Chinese Center for Diseases Control and Prevention (Chinese Center for Tropical Diseases Research), Shanghai, China; ^2^National Health Commission of the People’s Republic of China (NHC) Key Laboratory of Parasite and Vector Biology, Shanghai, China; ^3^World Health Organization (WHO) Collaborating Center for Tropical Diseases, Shanghai, China; ^4^National Center for International Research on Tropical Diseases, Shanghai, China; ^5^School of Global Health, Chinese Center for Tropical Diseases Research, Shanghai Jiao Tong University School of Medicine, Shanghai, China; ^6^School of Basic Medical Sciences and Forensic Medicine, Hangzhou Medical College, Institute of Parasitic Diseases, Hangzhou, China

**Keywords:** *Plasmodium vivax*, Duffy binding protein, genetic diversity, natural selection, China-Myanmar border

## Abstract

Malaria incidence has declined dramatically over the past decade and China was certified malaria-free in 2021. However, the presence of malaria in border areas and the importation of cases of malaria parasites are major challenges for the consolidation of the achievements made by China. *Plasmodium vivax* Duffy binding protein (PvDBP) performs a significant role in erythrocyte invasion, and is considered a promising *P. vivax* vaccine. However, the highly polymorphic region of PvDBP (PvDBP-II) impedes the development of blood-stage vaccine against *P. vivax*. In this study, we investigated the genetic diversity and natural selection of PvDBP-II among 124 *P. vivax* isolates collected from the China-Myanmar border (CMB) in Yunnan Province, China, during 2009–2011. To compare genetic diversity, natural selection, and population structure with CMB isolates, 85 *pvdbp-*II sequences of eastern Myanmar isolates were obtained from GenBank. In addition, global sequences of *pvdbp*-II were retrieved from GenBank to establish genetic differentiation relationships and networks with the CMB isolates. In total, 22 single nucleotide polymorphisms reflected in 20 non-synonymous and two synonymous mutations were identified. The overall nucleotide diversity of PvDBP-II from the 124 CMB isolates was 0.0059 with 21 haplotypes identified (*Hd* = 0.91). The high ratio of non-synonymous to synonymous mutations suggests that PvDBP-II had evolved under positive selection. Population structure analysis of the CMB and eastern Myanmar isolates were optimally grouped into five sub-populations (*K* = 5). Polymorphisms of PvDBP-II display that CMB isolates were genetically diverse. Mutation, recombination, and positive selection promote polymorphism of PvDBP-II of *P. vivax* population. Although low-level genetic differentiation in eastern Myanmar was identified along with the more effective malaria control measures, the complexity of population structure in malaria parasites has maintained. In conclusion, findings from this study advance knowledge of the understanding of the dynamic of *P. vivax* population, which will contribute to guiding the rational design of a PvDBP-II based vaccine.

## Introduction

Malaria, a common and life-threatening infectious disease, affects 2.29 billion people worldwide. *Plasmodium vivax* infected an estimated 5.3 million people in 2019, with most of the cases concentrated in Southeast Asia (WHO, 2020). The unique biology of *P. vivax* and repeated emergences of antimalarial resistance to vivax infections make this malaria parasite more difficult to control and eliminate than *P. falciparum* (Price et al., 2007). The Greater Mekong Subregion (GMS) in Southeast Asia has committed to achieve the goal of malaria elimination by 2030 (WHO, 2016). The member countries of GMS are Laos, Vietnam, Myanmar, Thailand, Cambodia, and China. Myanmar has the highest malaria burden in the GMS, with a major challenge for disease control and elimination, especially to neighboring countries such as China (Yunnan and Guangxi Provinces) and Thailand (Lo et al., 2017; Brashear et al., 2020). The China-Myanmar border (CMB) was once the area where the burden of malaria was highest in the world. In 2010, China has formulated the “Action plan of China malaria elimination (2010–2020)” and decided to accomplish the target of eliminating malaria across the country by 2020 (Feng et al., 2016). Malaria has substantially decreased over the past 10 years. Although China has been certified malaria-free in 2021, the country is still cautious about imported cases and malaria retransmission. In addition, malaria control in the CMB area is particularly a challenge owing to the high prevalence of malaria in Myanmar, resulting in continuous transmission and infection (Feng et al., 2020). Thus, cases of imported malaria pose a threat for sustainable malaria-free status within the borders of China. The development of an effective malaria vaccine is therefore a research priority for integrated malaria control. One of the main barriers for vaccine design is the antigenic diversity of candidate genes (Takala and Plowe, 2009) and it is essential to identify the determinants of antigenic variation in malaria-endemic areas. Meanwhile, with the progress in the implementation of malaria elimination measures, it is also reasonable to speculate whether there was a change in *P. vivax* population structure.

*Plasmodium vivax* invasion depends on a specific receptor-ligand interaction between the parasite and host red blood cells. The *P. vivax* Duffy binding protein (PvDBP) binds to the corresponding receptor Duffy antigen receptor for chemokines (DARC) to form a tight junction that is vital for merozoite invasion. Therefore, PvDBP is increasingly becoming an attractive vaccine molecule against vivax malaria. It could induce strong acquired immune responses in subjects from vivax endemic areas, and more importantly, antibodies against the domain II of PvDBP (PvDBP-II) block the interaction with DARC that prevent merozoite invasion from human erythrocytes (Grimberg et al., 2007; Longley et al., 2017). The PvDBP is a 140-kDa protein characterized by a functionally conserved cysteine-rich region, divided into seven different regions (Wertheimer and Barnwell, 1989). The key erythrocyte binding sites of PvDBP are mapped to a 170-amino acid stretch with cysteine regions (C4–C7) (Chootong et al., 2010). The PvDBP-II is highly polymorphic compared to the rest of the region. Genetic analysis from different geographical *P. vivax* isolates demonstrated that this region is under positive selection, with polymorphic residues varying by geographic region (Ampudia et al., 1996; Xainli et al., 2000; Ju et al., 2013; Chootong et al., 2014; Almeida-de-Oliveira et al., 2020). Despite PvDBP-II presenting a promising vaccine antigenic, diversity may affect the immune recognition and host immune response which may reflect that polymorphisms of PvDBP-II help in immune evasion mechanism (Cole-Tobian and King, 2003). Knowledge of the antigenic variation and natural selection of PvDBP-II is practical significance to fully comprehend the epidemiology of potential vaccine candidates and develop new interventions. Therefore, understanding the nature of genetic polymorphism within PvDBP-II in isolates from different geographic regions is important for developing a broadly reactive DBP-II-based protective vaccine against vivax malaria.

In this study, we collected 124 *P. vivax* samples from the CMB area in Yunnan Province of China at different times to analyze genetic diversity, natural selection, and the dynamic change with the population structure of PvDBP-II in CMB isolates. In addition, the association between CMB and eastern Myanmar *pvdbp-*II sequences were compared.

## Materials and Methods

### Ethics Statement

This study was conducted according to the principles expressed in the Declaration of Helsinki. Before blood collection, the study protocol and potential risks and benefits were explained to the participants, and written informed consent was obtained from each participant. Blood was collected following institutional ethical guidelines reviewed and approved by the Ethics Committee at the National Institute of Parasitic Diseases, Chinese Center for Disease Control and Prevention.

### Blood Samples, DNA Extraction, and PCR Amplification

**FIGURE 1:**
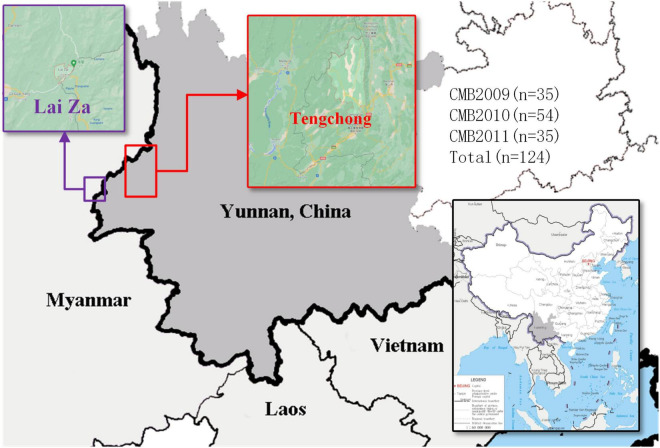
The map of *P. vivax* samples collection. Samples collection was made in Tengchong, marked in red rectangle (China-Myanmar border in Yunnan Province of China).

Blood samples were obtained from patients (who met the following inclusion criteria: aged from 18 to 65 years old and diagnosed positive for *P. vivax* infection by microscopic examination of blood smear) from the CMB area in Yunnan Province of China, during 2009–2011 (Figure 1). In total, we collected 124 *P. vivax* samples distributed as follows: 35 in 2009, 54 in 2010, and 35 in 2011. The QIAamp DNA mini kit (Qiagen, Germany) was used to extract genomic DNA as reported by previous methods (Chen et al., 2017). The *pvdbp*-II genes of CMB isolates were amplified by PCR using the following specific primers: *pvdbp*-II-F (5′-TGATAGTAAAACTGATAACGG-3′) and *pvdbp*-II-R (5′-TCTGATTTCCATTTTGACCAT-3′). The PCR reaction system was composed of 14 μl ddH_2_O, 5 × PrimeSTAR GXL buffer, 2.5 mM dNTP, 10 nM of each primer, 25 U/μl PrimerSTAR GXL Polymerase, and 2.5 μl genomic DNA. The cycling parameters for PCR amplification was performed using the following conditions: 98°C for 5 min, 35 cycles at 98°C for 10 s, 60°C for 15 s, 68°C 1.5 min, followed by extension at 68°C for 5 min. PCR products were examined by 1% agarose and sent to Beijing Genomics Institution (BGI, Shenzhen, China) for sequencing. The sequences have been deposited in the GenBank database under the accession numbers MZ765947-MZ766070.

### Genetic Diversity and Natural Selection of Polymorphic Region of PvDBP

To estimate genetic diversity and natural selection of PvDBP-II, the polymorphism between the CMB within-population isolates in 2009–2011 and eastern Myanmar (Laiza, Kachin State) *P. vivax* isolates in 2016 was compared. Sequences of the *pvdbp*-II from eastern Myanmar (*n* = 85, MN233489-MN233573) were obtained from GenBank (Hu et al., 2019). The *pvdbp*-II of Salvador I (DQ156512) was used as a reference sequence. Alignments of all sequences were performed using the CLUSTAL W method in MEGA 7.0 (Kumar et al., 2016). Genetic diversity of *pvdbp*-II was analyzed by using DnaSP 5.0 program (Rozas et al., 2003). Nucleotide diversity (π), number of haplotypes (*H*), haplotype diversity (*Hd*), number of segregating sites (S), and mean value of nucleotide differences (K) were calculated to analyze the genetic diversity of *pvdbp*-II. Neutrality of evolution was then detected by applying DnaSP 5.0 to calculate the rates of non-synonymous (dn) to synonymous (ds) mutations. If selective pressure is neutral, the expected value of the dn/ds is 1. However, if non-synonymous mutation is detrimental, the studied population is under purifying selection and the dn/ds is <1. If non-synonymous mutation is beneficial, the dn/ds > 1, and it is under positive selection. Significance was assessed by applying the *Z*-test using MEGA 7.0. In addition, Tajima’s D test was also performed to test neutrality with DnaSP. Tajima’s D test estimates the genetic variation within-population; namely, it calculates the number of segregating sites (θ) and the average number of nucleotide (π) differences estimated from pairwise comparison to test departures from the neutral theory of evolution, such as directional selection or balancing selection (Tajima, 1989).

### Recombination and Linkage Disequilibrium of Polymorphic Region of PvDBP

The recombination parameter (R: which includes the effective population size and probability of recombination between adjacent nucleotides per generation) and the minimum number of recombination events (Rm) were determined using DnaSP.5.0. Recombination events were evaluated by using ZZ statistic and Rm. Linkage disequilibrium (LD) between different polymorphic sites was computed in terms of the R^2^ index using DnaSP for the PvDBP-II of CMB isolates. R^2^ for each pair of genetic polymorphisms was plotted on heatmap graphics using the “LD heatmap” package (Rozas et al., 2001; Shin et al., 2006). LD was evaluated using the Zns statistic which represents the average of R^2^ overall pairwise comparisons.

### Genetic Differentiation, Haplotype Network, and Population Genetic Structure Analysis of Polymorphic Region of PvDBP

To estimate the degree of genetic differentiation of the *pvdbp*-II in global isolates, we download additional *pvdbp-II* sequences from GenBank, including Asia: India (FJ491142.1-FJ491241.1), Iran (EU860429.1-EU860435.1, KF791931.1-KF791925.1), and Thailand (EF379127.1-EF379132.1, EF368159.1-EF368164.1); Oceania: Papua New Guinea (AF289480-AF289483, AF289636-AF289647); Africa: Sudan (MG805621.1-MG805629.1); and South America: Brazil (EU812841.1-EU812845.1; EU812866.1-EU812873.1) (Cole-Tobian et al., 2002; Gosi et al., 2008; Babaeekhou et al., 2009; Sousa et al., 2010; Valizadeh et al., 2014; Hoque et al., 2018). All sequences were aligned and cut to 671 bp by MEGA7.0. Arlequin3.5 with analysis of molecular variance (AMOVA) was used to evaluate fixation (F_ST_) (Excoffier and Lischer, 2010). To establish a genetic relationship among the CMB, eastern Myanmar, and other countries’ haplotypes of PvDBP-II, the haplotype network was analyzed using NETWORK ver. 102200 with the Median-Joining method (Bandelt et al., 1999). The software STRUCTURE (ver. 2.3.4) was used to determine the genetic structure of *P. vivax* population in the CMB and eastern Myanmar (Pritchard et al., 2000). The Bayesian method was used to identify the optimum number of clusters (K). The admixture model for values *K* = 3–9 was run, each with a burn-in period of 10,000 steps and then 100,000 Markov Chain Monte Carlo (MCMC) iterations. The best-fit number of grouping was evaluated using delta K (ΔK) in the STRUCTURE HARVESTER.

## Results

### Haplotype Variation and Nucleotide Diversity of Polymorphic Region of PvDBP From Different *P. vivax* Isolates

**FIGURE 2:**
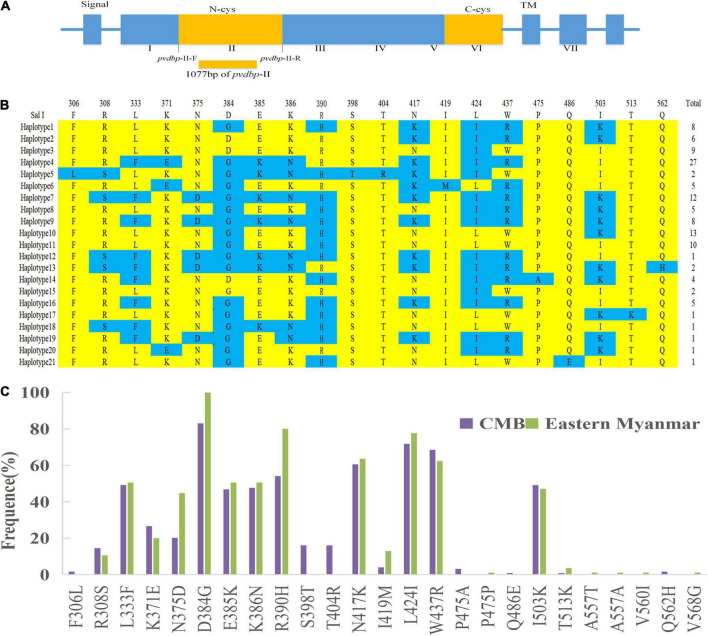
Sequence and polymorphism of PvDBP-II in *P. vivax* CMB isolates. **(A)** The structure of *Plasmodium vivax* Duffy binding protein. **(B)** The changes in amino acid sequences. Polymorphic amino acids are listed for each haplotype. Amino acid residues identical to those of the reference sequence, *SaI* I (DQ156512), are marked in yellow. The dimorphic amino acid changes are marked in blue. Total number of sequences for each haplotype is listed in right panel. **(C)** Frequencies of amino acid changes found in PvDBP-II among CMB and eastern Myanmar isolates.

A total of 124 samples from the CMB were successfully amplified and sequenced for the *pvdbp*-II (1077 bp) which corresponds to amino acid positions at 222–580 of the Salvador I reference (Figure 2A). The 124 CMB samples had 22 single nucleotide polymorphisms (SNPs) of which two and 20 were synonymous and non-synonymous (Table 1) polymorphic codons, respectively (nine occurred at the first base of the codon, five at the second, and eight at the third base of the codon), causing significant amino acid changes in protein level. Analysis of the deduced amino acid sequences classified them into 21 haplotypes (Haplotypes 1–21) with amino acid changes at 20 positions, in which all were dimorphic. Haplotype 4 was predominant (*n* = 27, 21.8%) among CMB isolates (Figure 2B).

**TABLE 1:** Genetic diversity and natural selection of *pvdbp*-II in China-Myanmar border and eastern Myanmar isolates.

Population	n	S	η	*H*	*Hd*	π	k	Tajima’s D	*P*-value	dn	ds	dn/ds	*P-*value
CMB2009	35	19	19	13	0.92	0.006	5.976	0.9943	*P* > 0.10	0.007	0.0024	2.962	*P* < 0.05
CMB2010	54	17	17	14	0.9	0.0056	5.584	1.5326	*P* > 0.10	0.0066	0.0019	3.474	*P* < 0.05
CMB2011	35	20	20	14	0.91	0.006	6.037	0.823	*P* > 0.10	0.0071	0.0022	3.227	*P* < 0.05
CMB2009-2011	124	22	22	21	0.91	0.0059	5.813	1.2158	*P* > 0.10	0.0068	0.0021	3.238	*P* < 0.05
Eastern Myanmar	85	20	21	16	0.85	0.0058	5.784	1.1337	*P* > 0.10	0.0064	0.0038	1.684	*P* < 0.05

*CMB, China-Myanmar border; n, number of samples; S, number of segregating sites; η, the total number of mutations; H, number of haplotypes; Hd, haplotype diversity; π, nucleotide diversity; k, the average of nucleotide differences; dn, the rates of non-synonymous substitutions; ds, the rates of synonymous substitutions.*

Nineteen of the 20 non-synonymous changes were previously identified, whereas the remaining one change (Q562H, 1.6%) was unique in CMB isolates, which was not hitherto reported. D834G (83.1%), L424I (71.8%), and W437R (68.6%) SNPs were the most frequent. Comparison of the amino variants observed in PvDBP-II revealed that CMB isolates had a similar pattern compared to eastern Myanmar isolates.

Although eastern Myanmar isolates showed similar amino changes compared to CMB isolates, six variants (F306L, S398T, T404R, P475A, Q486E, and Q562H) detected in the CMB isolates were not found in eastern Myanmar isolates (Figure 2C).

**FIGURE 3:**
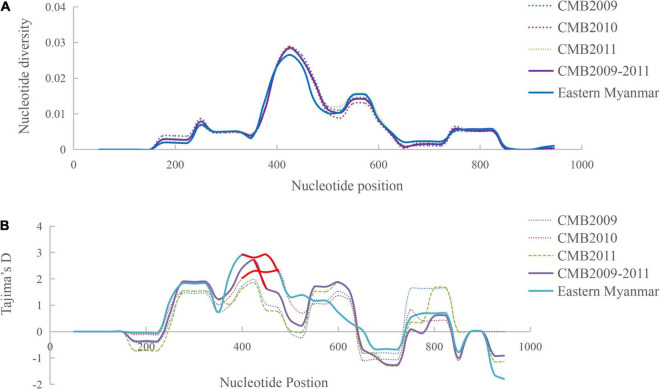
Polymorphism and Tajima’s D tests of *pvdbp*-II sequences from CMB and eastern Myanmar isolates. **(A)** Sliding window plots of nucleotide diversity per site (π) comparing the level of genetic diversity at *pvdbp*-II. **(B)** Sliding window plots of Tajima’s D for *pvdbp*-II. Statistical significant sites are marked with red lines.

Analysis of polymorphism within the 124 *pvdbp*-II CMB isolates revealed lower nucleotide diversity (π = 0.0059) and higher haplotype diversity (*Hd* = 0.91) (Table 1). A sliding window plot of π with a window of 100 bp and step size of 25 bp revealed values for all CMB isolates ranging from 0.000 to 0.028. The highest peak of nucleotide diversity within the *pvdbp*-II of CMB isolates was between nucleotide positions 360 and 480. Nucleotide diversity of CMB isolates in 2009, 2010, and 2011 was 0.0060, 0.0056, and 0.0058, respectively, which collectively was a little higher than that of eastern Myanmar isolates (Figure 3A).

### Natural Selection of Polymorphic Region of PvDBP From Different *P. vivax* Isolates

To determine whether natural selection contributed to the generation of diversity in *pvdbp*-II within the China-Myanmar *P. vivax* population, we calculated the rate of non-synonymous (dn) to synonymous (ds) mutations. The rate of non-synonymous to synonymous substitution (dn/ds) for PvDBP-II for all the 124 CMB isolates was 3.238 (with rates of 2.962, 3.4736, and 3.227 for isolates of 2009, 2010, and 2011, respectively), suggesting that a positive natural selection might occur in the PvDBP-II of CMB isolates. Furthermore, the overall Tajima’s D value for PvDBP-II was 1.2158 for all the 124 CMB isolates (with values of 0.9943, 1.5326, and 0.8230 for isolates of 2009, 2010, and 2011, respectively) (Table 1 and Figure 3B). In addition, by sliding the window to detect specific regions under selection, we confirmed that significant positive Tajima’s D values (*P*<0.05) were found in the 1,115–1,185 bp region from the CMB 2009 isolates and the 1,130–1,165 bp region from all the CMB isolates in 2009–2011. Such an informative result suggests a positive balancing selection for PvDBP*-*II in the CMB population. Moreover, eastern Myanmar isolates were also found under positive balancing selection.

### Recombination

**FIGURE 4:**
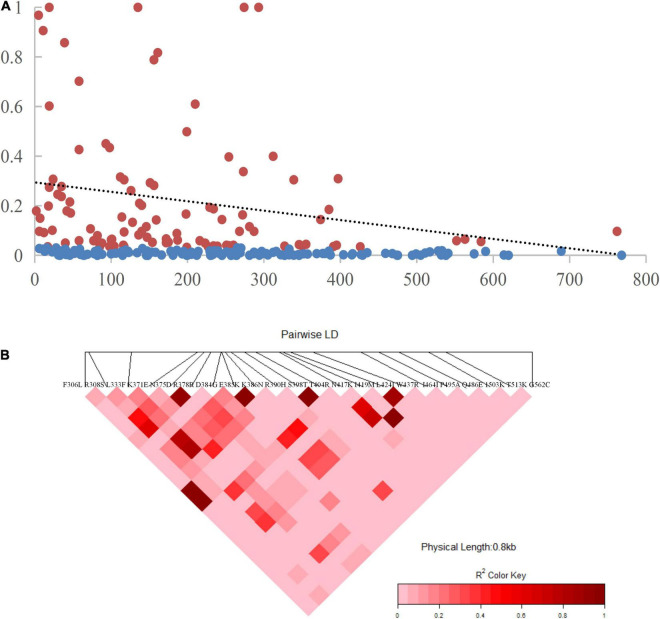
Linkage disequilibrium (LD) plot showing non-random association between nucleotide variants among 124 CMB *P. vivax* isolates at different polymorphic sites. **(A)** The significant LD sites detected by Fisher’s exact test are marked red pots and others are marked blue pots. The dashed line represents the regression. **(B)** R^2^ for each pair of genetic polymorphisms of CMB isolates.

The recombination events (Rm) between adjacent polymorphic sites of the 124 CMB isolates was 7 (with values of 6, 5, and 7 for isolates of 2009, 2010, and 2011, respectively) (Table 2). In 124 CMB isolates, we found N375D/R378R, E385K/K386N, S398T/T404R, and L424I/W437R on high LD levels. The value of ZZ, Zns, and the decline of R^2^ with an increasing distance between the pairs of nucleotide sites suggest that intragenic recombination may also contribute to the diversity of PvDBP-II in CMB isolates (Figure 4).

**TABLE 2:** Linkage disequilibrium and recombination of *pvdbp*-II in China-Myanmar border and eastern Myanmar isolates.

Population	Rm	Zns	ZZ
CMB2009	6	0.151	0.118
CMB2010	5	0.150	0.077
CMB2011	7	0.138	0.103
CMB2009-2011	7	0.104	0.112
Eastern Myanmar	5	0.166	0.077

*CMB, China-Myanmar border; Rm, minimum number of recombination events.*

### Genetic Differentiation, Population Structure, and Clustering of Polymorphic Region of PvDBP Haplotypes

The level of genetic differentiation of *pvdbp*-II was estimated by F_ST_ values. In general, the value of F_ST_ (0.05–0.15) is poor differentiation, F_ST_ (0.15–0.25) is moderate differentiation, and F_ST_ >0.25 is great differentiation (Balloux and Lugon-Moulin, 2002). The CMB isolates, eastern Myanmar isolates, and Thailand isolates showed a poor genetic differentiation with the values of F_ST_ being 0.0296 and 0.0568, respectively. The high level of genetic differentiation was found between CMB isolates Sudan and Papua New Guinea (PNG) (the values of F_ST_ were 0.2517 and 0.4598, respectively) (Table 3).

**TABLE 3:** Estimation of genetic differentiation (Fst) of the *pvdbp*-II among other geographical populations.

Population	Brazil	CMB	India	Iran	Eastern Myanmar	PNG	Sudan	Thailand
Brazil (*n* = 13)	0.0000							
CMB (*n* = 124)	0.2764	0.0000						
India (*n* = 100)	0.1566	0.0787	0.0000					
Iran (*n* = 122)	0.1281	0.0816	0.0128	0.0000				
Eastern Myanmar (*n* = 85)	0.3348	0.0296	0.12387	0.1374	0.0000			
PNG (*n* = 16)	0.5709	0.4598	0.4157	0.4338	0.4787	0.0000		
Sudan (*n* = 10)	0.6353	0.2517	0.2775	0.2974	0.3143	0.7044	0.0000	
Thailand (*n* = 12)	0.2498	0.0568	0.0817	0.1083	0.0726	0.4150	0.3421	0.0000

*CMB, China-Myanmar border; PNG, Papua New Guinea.*

**FIGURE 5:**
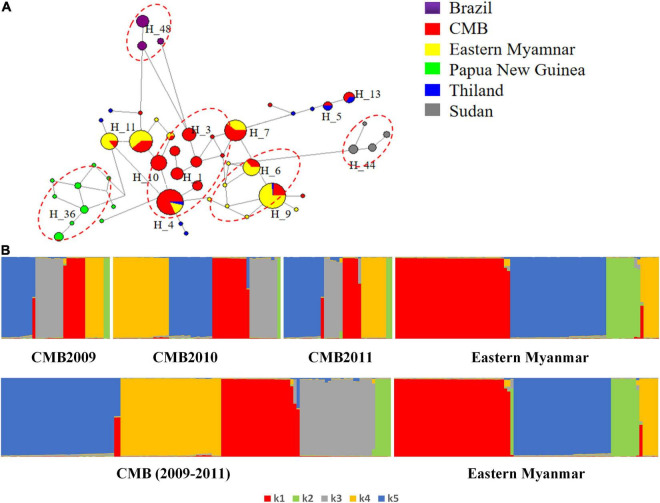
STRUCTURE and Network analysis of PvDBP-II haplotypes. **(A)** The haplotype network shows the relationships among 50 haplotypes present in sequences of 260 isolates. **(B)** STRUCTURE analysis of the full set of variation loci from all isolates. Cluster for each isolate was assessed to an optimized cluster value of *K* = 5.

Collectively, a total of 50 haplotypes were identified from 260 *pvdbp*-II sequences of the CMB, eastern Myanmar, Sudan, Thailand, and PNG isolates, with 26 singleton haplotypes (observed only once). The haplotypes network could be roughly grouped into five major clusters and some small scattered groups (Figure 5A). Five major clusters based on geographical distribution were CMB isolates, Myanmar mix isolates, PNG isolates, Brazil isolates, and Sudan isolates. In the network analysis, the haplotype prevalence ranged from 0.38 to 12.69%. Haplotype 4 was shared among CMB, eastern Myanmar, and Thailand. Haplotype 7 and 11 were shared among CMB isolates and eastern Myanmar isolates with relatively high frequencies. H5 and 13 were shared with CMB and Thailand isolates. Whilst H1, H2, H3, H8, H10, H12, H15, H17, H18, H19, and H20 only existed in CMB isolates, H10 was the dominant haplotype.

With regard to the population structure of the haplotype of *pvdbp*-II from the CMB and eastern Myanmar isolates, STRUCTURE analysis showed a clear distribution of haplotypes and demonstrated multiple sub-populations. The haplotypes were optimally grouped into five sub-populations *(K* = 5; Figure 5B). Three sub-populations of malaria parasites from CMB in 2009–2011 had admixed haplotypes which showed a similar distribution. Compared with CMB populations, the eastern Myanmar population was composed of four sub-populations and a significant decrease (*P* < 0.05) was noted in the K3 sub-population, while a significant increase (*P* < 0.05) was found in the proportion of the K1 sub-population (Figure 5B).

## Discussion

Due to vector species, host genetics, and environmental factors, *P. vivax* populations harbor different genetic diversities under different geographies (Arnott et al., 2012). The high level of diversity within *Plasmodium* antigens is a major challenge for effective malaria vaccines. Compared to merozoite surface proteins (MSPs) and circumsporozoite protein (CSP), PvDBP-II bears less polymorphism (Shen et al., 2015). Hence, PvDBP-II is considered as the primary fragment of candidate antigen for vaccine against malaria. Investigation on polymorphism and population structure of PvDBP-II may provide deeper criteria for the selection of vaccine candidates. In the present investigation, we analyzed the genetic diversity and molecular evolution of PvDBP-II in the CMB populations in Yunnan Province of China and compared them to that of eastern Myanmar populations. From the 124 CMB *P. vivax* isolates collected during 2009–2011, a total of 22 SNPs were divided into 21 distinct haplotypes. Overall, haplotype diversity (*Hd*) from CMB was 0.91, which showed similar diversity in isolates from other endemic areas such as Brazil, Myanmar, Thailand, and Sudan.

Based on amino acid variants, 22 polymorphic residues were detected in the CMB isolates. Besides one unique mutation (Q562H), the remaining mutations were previously reported from global *P. vivax* isolates. The seven most common mutations (K371E, D384G, R390H, N417K, L424I, W437R, and I503K) of PvDBP-II in global *P. vivax* populations were also found among CMB isolates. D384G (83.1%), L424I (71.8%), and W437R (68.6%) were the most prevalent polymorphic variants in CMB isolates. The frequency of SNPs obtained in this study was similar to that from Myanmar, Sudan, and Thailand, whereas it was smaller than that from PNG and Sri Lanka (Premaratne et al., 2011; Ju et al., 2012). Comparison of SNPs’ frequencies identified in this study to those from other areas where vivax malaria is endemic revealed that most observed SNPs in CMB isolates showed different frequencies than what has been previously reported. This information may reflect the intensity of malaria transmission in different areas and the diversity of PvDBP-II may vary by geographic area. Three residues, namely N417K, W437R, and I503K, forming an important discontinuous epitope in PvDBP-II, were described as the main target for binding inhibitory antibodies against erythrocyte binding (Chootong et al., 2010; McHenry et al., 2011). In this study, N417K (60.5%), W437R (68.6%), and I503K (49.2%) were detected and all the three variants were found simultaneously in 18 isolates. Such an informative finding suggests that single or combined sites polymorphisms affect the efficiency of PvDBP-II inhibitory antibodies against invasion and help the parasites evade host immune attacks. All these mutations might generate PvDBP-II-based vaccine-resistant parasite isolates.

The pattern of polymorphism observed in part of PvDBP-II revealed this region under positive balancing selection confirmed by Tajima’s D test. The direction of Tajima’s D is potentially informative of the population evolution. The positive Tajima’s D test of the evolutionary force on the populations indicated CMB population size reduction. Previous studies have found PvDBP-II under a strong selection. The high rates of non-synonymous to synonymous mutations reflected a positive selection promoting greater polymorphism, which may allow the evasion of host immune selection independently of the geographical distribution (Li et al., 2020). In this study, the rate of non-synonymous to synonymous mutations indicated that the PvDBP-II of CMB isolates was also under positive selection. Therefore, the high value of non-synonymous to synonymous mutations and positive Tajima’s D support the theory that an increase in polymorphisms within the *pvdbp*-II of CMB isolates results in escape of host immune attacks.

Intragenic recombination is an important factor for genetic diversity of *P. vivax* isolates, which could increase variation in the PvDBP-II. LD decreased as nucleotide distance increased, suggesting that recombination has been taking place in *pvdbp*-II among CMB isolates. Recombination was confirmed by using ZZ tests, which contributed to the diversity. Such an interpretation aligns onto similar findings from previous reports from other regions where *P. vivax* is endemic, such as Myanmar, Korea, and Sri Lanka. Despite *P. vivax* having high diversity, significant LD may reflect multispatial infections within a population. The relatively higher number of recombination events in the CMB than in eastern Myanmar isolates might reflect the existence of *P. vivax* infection in mixture populations. With the progress in control programmes for malaria elimination, the number of recombination events might correlate with a decrease in *P. vivax* transmission.

*P. vivax* has become the most dominant parasite in the GMS region. Better understanding of transmission dynamics and population structure is essential to develop appropriate interventions for malaria elimination (Li et al., 2020). Myanmar border malaria is a major source of disease transmission and infection in the CMB in Yunnan Province. It was introduced by the highly mobile human populations across the border. The low F_ST_ values were observed between CMB isolates, eastern Myanmar and Thailand isolates, reflecting human mobility which facilitated gene flow between parasite populations of GMS region. STRUCTURE and network analyses further confirmed the relationship between the CMB, Myanmar, and other populations for PvDBP-II. This study demonstrated that the proportion of parasite populations decreased significantly with the time and geographic location difference. Our results displayed a more complex population structure than that of GMS *P. vivax* population which was only composed of four clusters based on PvDBP-II (Hu et al., 2019). Importantly, eastern Myanmar isolates lacked the K3 sub-population that existed in CMB isolates, indicating that no *P. vivax* population was imported from CMB to Myanmar. In 2010, China initiated the “Action plan of China malaria elimination (2010–2020).” Fighting malaria in the CMB has been a remarkable success, with cases having declined significantly, moving from control stage to elimination stage (Hewitt et al., 2013). Hence, in 2016, there was no doubt that CMB isolates or CMB imported cases did not exist in eastern Myanmar. The haplotype network revealed that *P. vivax* populations were highly heterogenetic and dynamics of malaria transmission differed in different areas (Cui et al., 2012). Interestingly, the network analysis of PvDBP-II showed that most of the prevalent haplotypes were shared among CMB and Myanmar isolates. In addition, some haplotypes were unique in CMB isolates which may associate with the mutations and the change of population structure. Such a finding advanced our knowledge of the parasite population dynamics in this region for the rational design of effective interventions to block disease transmission.

Designing an effective vaccine against *P. vivax* requires antigens with limited genetic diversity. Although *pvdbp*-II of CMB isolates bear some diversity, it is noteworthy that the prevalent Haplotype 4 (12.7%) was shared with multiple populations, which may present an attractive point for vaccine development.

## Conclusion

This investigation provided the description of genetic polymorphism and natural selection of *pvdbp*-II in the CMB of Yunnan Province during 2009–2011. Results indicated that PvDBP-II was genetically diverse in the CMB. Also, findings from this study further confirmed that mutations, natural selection, and recombination might increase and sustain evasion of host immunity. With the remarkable progress made in malaria control at the CMB, this population showed specific structure and temporal differentiation. These findings provide new insights into *P. vivax* population structure and evolution in the CMB, and more importantly, consolidate the basis for rational development of an effective blood-stage malaria vaccine based on antigen variation and dominant haplotype.

## Data Availability Statement

All materials and data supporting these findings are contained within the manuscript. The sequences have been deposited in the GenBank database under the accession numbers MZ765947–MZ766070 for the China-Myanmar border isolates in Yunnan Province of China.

## Ethics Statement

The studies involving human participants were reviewed and approved by the Ethics Committee at National Institute of Parasitic Diseases, Chinese Center for Disease Control and Prevention. Written informed consent to participate in this study was provided by the participants’ legal guardian/next of kin.

## Author Contributions

T-QS, J-HC, BZ, and YW conceived, wrote the manuscript, and designed the experiments. T-QS, S-BC, Y-BC, BX, and YW performed the experiments. T-QS, H-MS, KK, and YW analyzed the data. S-BC, KK, Y-BC, BX, and YW contributed the reagents, materials, and analysis tools. All authors contributed to the article and approved the submitted version.

## Conflict of Interest

The authors declare that the research was conducted in the absence of any commercial or financial relationships that could be construed as a potential conflict of interest.

## Publisher’s Note

All claims expressed in this article are solely those of the authors and do not necessarily represent those of their affiliated organizations, or those of the publisher, the editors and the reviewers. Any product that may be evaluated in this article, or claim that may be made by its manufacturer, is not guaranteed or endorsed by the publisher.
